# Deubiquitinase USP38 Stabilizes PLK1 Expression to Boost DNA Damage Repair in Ovarian Cancer

**DOI:** 10.1002/kjm2.70197

**Published:** 2026-03-19

**Authors:** Yuan Ma, Ying Li, Kai‐Li Li

**Affiliations:** ^1^ Department of Gynecology Anyang Tumor Hospital Anyang China

**Keywords:** deubiquitination, DNA damage repair, ovarian cancer, PLK1, USP38

## Abstract

Ovarian cancer (OC) is one of the most prevalent and severe gynecological malignant tumors. DNA damage repair (DDR) is essential in maintaining genome stability. This study aims to investigate the effects and mechanisms of USP38 and PLK1 on DNA damage repair and malignant behavior in OC cells. Bioinformatics analysis was conducted to assess the expression of USP38 and PLK1 in OC tissues and predict downstream target proteins of USP38 and potential deubiquitination sites of target proteins. Expression levels of USP38 and PLK1 were analyzed by qPCR and WB. Cell proliferation ability was assessed by EdU experiment assay and colony formation assay. Apoptosis level was analyzed by TUNEL assay. The CO‐IP assay was applied to assess the binding of USP38 and PLK1. The ubiquitination experiment was employed to evaluate the ubiquitination of PLK1 by USP38. The comet assay was utilized to analyze DNA damage in cells. USP38 and PLK1 were highly expressed in OC. The binding of USP38 to PLK1 mediated the deubiquitination of PLK1 and stabilized the protein level of PLK1. Silencing USP38 reduced the proliferation ability of OC cells and elevated the apoptosis rate of cancer cells. Mechanically, silencing USP38 decreased the expression level of PLK1 protein, repressing DDR in cancer cells. Deubiquitinase USP38 binds to PLK1 to stabilize PLK1 expression, facilitating DDR in OC cells and enhancing cancer cell proliferation, thereby reducing the apoptosis rate of cancer cells.

## 1 Introduction

Ovarian cancer (OC) is a very fatal and aggressive gynecological malignancy, ranking as the eighth most prevalent cancer and the fifth most fatal cancer in women throughout the world [1]. The incidence rate of OC is reported as 3.4%, with a mortality rate of 4.7%. Every year, more than 300,000 women are diagnosed with OC, and approximately 150,000 women die from it [2]. OC certainly poses a daunting threat to women's health and survival. The current standard treatment for OC includes surgical treatment and platinum‐based chemotherapy [3]. Targeted therapy and immunotherapy are commonly used in OC treatment [4, 5]. However, due to drug resistance and easy recurrence, OC often leads to about half of patients experiencing recurrence within 2 years after treatment, with no substantial improvement made in the survival rate [6, 7]. In addition, due to the hidden physiological and anatomical location of the ovary, OC lacks strong early detection indicators, which leads to the missing of the optimal treatment opportunity for patients, thus exacerbating patients' dismal prognosis [8]. Therefore, innovating potential biomarkers of OC, probing into the related molecular mechanisms regulating the malignant behavior of OC, and refining the adverse prognosis of OC patients have become research hotspots.

When DNA is attacked by endogenous and exogenous factors, it can cause self‐damage, resulting in instability in the genome. To protect the genome from damaging factors, the body has evolved DNA damage response systems, which involve several factors, including sensing DNA damage, signaling damage to cells, and recruiting downstream effector proteins. Under the joint action of these factors, cells will carry out DNA damage repair (DDR). Normally, DDR induces processes such as cell cycle arrest and activation of transcription programs. However, if the damage is severe, it activates processes such as cell death and aging and ultimately ceases the transmission of genetic information in damaged DNA [9, 10]. Inadequate repair of DNA damage can result in gene mutations or chromosomal aberrations, which can easily induce cancer [11]. DNA damage is proven to be linked with cancers such as gastric cancer (GC), colorectal cancer (CRC), prostate cancer, OC, and so forth [12, 13, 14, 15]. With further investigation on DNA damage, DNA damage response is found to be not only linked with cancer susceptibility but also affects the cancer progression and treatment efficacy [16]. For example, Saini et al. [17] suggested that silencing MMRN1 can dampen the cell viability, migration, and invasion ability of cancer cells in OC, thereby elevating the apoptosis rate, which is achieved by modulating the ability of cancer cells to repair DNA damage. In short, DNA damage response is a pivotal factor mediating the occurrence and development of cancer. The investigation of the relation and molecular mechanism between DNA damage and OC is expected to refine the poor prognosis of OC patients.

Ubiquitin‐specific protease 38 (USP38) is a deubiquitinase, belonging to the ubiquitin‐specific protease (USP) family, which can mediate the deubiquitination of substrate proteins [18]. It is well known that deubiquitinase‐mediated deubiquitination modification and ubiquitin ligase‐mediated ubiquitination modification play essential regulatory roles in human cancer and other diseases [19]. USP38 plays a role in various diseases such as asthma, severe malaria, emphysema, colon cancer, breast cancer, and lung cancer [20, 21, 22, 23]. For example, Wang et al. [24] asserted in a study on CRC that USP38 can directly interact with HMX3, mediating deubiquitination of HMX3 to stabilize its protein expression level. Elevated expression of HMX3 can repress the proliferation, migration, and invasion of CRC cells. They suggested that USP38 is a promising therapeutic target for CRC [24]. Although most scholars have pointed out that USP38 can mediate the malignant progression of cancer, the relationship between USP38 and OC remains unelucidated. Additionally, an in‐depth investigation of the regulatory mechanism of USP38 revealed that after USP38 binds to substrate proteins and mediates deubiquitination of substrate proteins, it can modulate cell DNA damage responses by stabilizing the protein level of substrate proteins, thereby influencing cell function [25]. In conclusion, USP38 plays an essential part in the malignant progression of tumors, but its relationship with OC and its relevant mechanisms are rarely reported, necessitating scholars to probe into this area to innovate new potential targets and methods for treating OC.

This study demonstrated that USP38 and PLK1 were highly expressed in OC. USP38 bound to PLK1, mediated deubiquitination of PLK1, and stabilized PLK1 protein levels. Silencing USP38 dampened the proliferation ability of OC cells and elevated the apoptosis rate of cancer cells, which can be explained mechanistically that USP38 silence lowered the level of PLK1 protein, thus hindering DDR in cancer cells. In other words, the USP38/PLK1 regulatory axis can modulate the proliferation and apoptosis of OC cells, serving as an essential factor influencing DDR in OC cells.

## 2 Materials and Methods

### 2.1 Bioinformatics Analysis

The mRNA expression data of OC were available for download from the Cancer Genome Atlas (TCGA) database (normal: 88, tumor: 379). The differential analysis of mRNA expression was conducted by using the *edgeR* package, with parameters set as |logFC| > 1 and FDR < 0.05. Based on the literature, USP38 was selected as the core gene for this work from numerous differentially expressed genes (DEGs). The differential mRNA expression of USP38 in OC samples and normal samples was analyzed using a *t*‐test (*p* < 0.05). By using the Ubibrowser database, we predicted downstream target proteins of USP38 and their possible binding sites to target proteins. The parameters in the Ubibrowser database were set as confidence score > 0.8 and the candidate proteins ranked in the top 20. Protein docking maps of PLK1 and USP38 were analyzed using Hdock. Gene set enrichment analysis (GSEA) was applied in the enrichment analysis of DEGs caused by the differential expression of PLK1.

### 2.2 Cell Culture

The normal human cell line used in this study was IOSE‐80 (catalog number Delf‐10500), and the human OC cell lines were Caov‐3 (catalog number BNCC101010), PA‐1 (catalog number BNCC100460), and SK‐OV‐3 (catalog number BNCC338639). IOSE‐80 cells were cultivated in the RPMI‐1640 complete medium. Cells and the medium were purchased from Hefei Wanwu Biotech Co. Ltd. (China). Caov‐3 cells were cultivated in the complete medium DMEM‐H. PA‐1 cells were in complete medium EMEM. SK‐OV‐3 cells were cultivated in a 5A medium. Cells and these media were purchased from BeNa Culture Collection (BNCC, China). During cell culture, 1% P/S double antibody was added to the culture medium and cultured in a constant temperature incubator (Thermo Fisher Scientific, USA) with a temperature of 37°C, a humidity of about 90%, and 5% CO_2_. When the relative cell number reached 80%–90%, the medium was aspirated and washed twice with phosphate‐buffered saline (PBS) buffer. Then, 1 mL of 0.25% trypsin (Gibco, USA) was introduced for 1 min for subculture. The cells underwent subculturing every 2–3 days.

Onvansertib was purchased from MedChemExpress (USA) as a highly selective PLK1 inhibitor. Cells were cultured with 50 nM Onvansertib for 24 h.

### 2.3 Cell Transfection

For cellular transfection, the pGPU6 plasmid was utilized for gene silencing, and the pEX‐1 plasmid for gene overexpression, both sourced from GenePharma (China). The silent gene was USP38, and the overexpressed gene was PLK1. The USP38 mutant (C454A/H857A/D918N) was cloned into the pcDNA3.1 vector (Invitrogen, USA). To ensure the success of the ubiquitination validation experiment, we transfected cells with the pCMV‐HA‐Ub plasmid, which was purchased from Abiowell Biotechnology Co. Ltd. All synthetically expressed plasmids were cell‐infected by utilizing Lipofectamine 3000 (ThermoFisher Scientific, USA) to obtain transiently expressed cell lines. In brief, cells were first cultured to 70%–90% confluence, then the reaction solution was prepared as required and mixed in proportion (1:1) into the cell culture medium. After 5 min of incubation at room temperature, we introduced the nucleic acid‐liposome complex, incubated the cells at 37°C for 2–4 days, and analyzed the transfected cells for subsequent experiments.

### 2.4 Quantitative Polymerase Chain Reaction (qPCR)

The TRIzol reagent kit (Invitrogen Life Technologies, USA) was implemented for the extraction of total RNA from cells. The subsequent reverse transcription into cDNA was facilitated by the PrimeScript RT reagent kit (Takara, Japan). qPCR analysis was then executed with SYBR Green (Nanjing Vazyme, China) on a CFX96 RealTime PCR System (Bio‐Rad, USA). The primer sequences were bought from Tsingke Biotechnology Co. Ltd. (China). The USP38 primer sequences were F: 5′‐GCAAGTATGACCCAAGCCCT‐3′ and R: 5′‐AGGAAAATCCTTTATTGCCTCCA‐3′. The PLK1 primer sequences were F: 5′‐GTGACGGCACTGAGTCCTAC‐3′ and R: 5′‐GCTCGCTCATGTAATTGCGG‐3′. The internal reference GAPDH primer sequences were F: 5′‐GAAGGTCGGAGTCAACGGAT‐3′ and R: 5′‐CCTGGAAGATGGTGATGGGAT‐3′. The RNA levels of the genes were normalized against the GAPDH RNA level, with the relative gene expression calculated using the 2^−ΔΔCt^ formula.

### 2.5 Western Blot (WB)

Total cell proteins were extracted with NP40 lysis buffer (Beyotime, China). Protein concentration was assessed using a BCA protein assay kit (Beyotime, China). Equal volumes of protein samples were loaded into a Precast mini‐protoean TGX gel (BioRad, USA) for electrophoresis. Following electrophoresis, proteins were transferred to a PVDF membrane, which was covered with the primary antibody and incubated overnight at 4°C after blocking with 5% skim milk for 1 h. Thereafter, the protein was rinsed with TBST and incubated with the secondary antibody for 4 h at room temperature. Another TBST washing preceded protein visualization with a high‐sensitivity ECL reagent kit (P0018M, Beyotime, China). Bands were imaged using a chemiluminescence imaging system. The primary antibodies included USP38 antibody (ab72244, Abcam, UK, 1:500), PLK1 antibody (ab189139, Abcam, UK, 1:1000), p‐CHK1 antibody (ab58567, Abcam, UK), γH2AX antibody (AP0687, ABclonal, China, 1:7000). The secondary antibody was goat anti‐rabbit IgG (ab205718, Abcam, UK, 1:5000). Each blotting was conducted at least three times. The GAPDH (ab9485) was purchased from Abcam (UK).

### 2.6 5‐Ethynyl‐2′‐Deoxyuridine (EdU) Experiment

The EdU experiment was employed to assess the proliferation of cells in each group. The EdU‐555 cell proliferation detection kit (catalog number C0075S) was purchased from Beyotime (China). In simple terms, we seeded cells of each group into a 96‐well plate (1 × 10^3^ cells/well), prepared various reaction solutions, and labeled, fixed, and stained the cells. Finally, after washing the cells with PBS, the fluorescence image of the cells was observed using a fluorescence microscope.

### 2.7 Colony Formation Assay

Under normal culture conditions, cells were seeded in a 6‐well plate (200 cells/well) and cultivated for 1 week. Upon the visible formation of colonies, the 6‐well plate was taken out, the medium was aspirated, and cells were fixed with 2 mL of 4% paraformaldehyde for 30 min. After that, the formaldehyde was poured off, and 2 mL of 0.1% crystal violet was introduced into each well for 15 min of staining. Subsequently, the crystal violet was rinsed away, and the colonies were enumerated with the aid of a computer.

### 2.8 TUNEL Assay

Apoptosis in each group was evaluated using the TUNEL assay. The TUNEL apoptosis detection kit (catalog number C1086) was purchased from Beyotime (China), and the specific operations were carried out strictly per the instructions. In simple terms, we inoculated cells from each group into a 96‐well plate (1 × 10^3^ cells/well) and immobilized the cells with 4% paraformaldehyde. After fixation, the cells were permeabilized as required. Next, the reaction solution was prepared according to the kit instructions. The cells were stained. Finally, the fluorescence images of the cells were analyzed under a fluorescence microscope after washing and resuspending the cells with PBS.

### 2.9 Co‐Immunoprecipitation (CO‐IP) Experiment

Cells from each treatment group were cultured for about 2 days and collected, with approximately 1 × 10^8^ cells collected from each treatment group. The collected cells were lysed on ice for 20–30 min using RIPA lysis buffer (Applygen Technology Co. Ltd., China). After lysis, a high‐speed centrifuge was applied for 10 min of centrifugation, and the resulting supernatant was collected. The supernatant was grouped into two groups (the Input group and the IP group). The IP group antibody was USP38 antibody (ab72244, Abcam, UK, 1:500). The Input group antibodies were USP38 and PLK1 (ab189139, Abcam, UK, 1:1000), with GAPDH as an internal reference (ab9485, Abcam, UK). Subsequently, the supernatants of each group were gathered and incubated with antibodies overnight at low temperatures by rotation. After incubation, the precipitate was collected by centrifugation for 1–2 min. Next, Dynabeads magnetic beads (Thermo Fisher Scientific, USA) were incubated with the supernatant at low temperature for 2 h, centrifuged for 2 min, and the protein on the magnetic beads was eluted for WB analysis.

### 2.10 Protein Binding Stability Verification Experiment

Two experiments were performed to verify the impact of USP38 on the stability of the PLK1 protein. The first one was to add the protein synthesis inhibitor CHX to the cell culture medium of each group and use WB to examine the protein expression levels of USP38 and PLK1 in cells of each group during different periods (0, 12, 24, and 48 h). The second one was to randomly divide the cells into two groups, with one group having PBS in the medium and the other group having the 26S proteasome inhibitor MG132 in the medium. We collected cells from each group after 12 h of cultivation and analyzed the protein expression levels of USP38 and PLK1 in each group of cells using WB. The WB experiment was conducted according to the steps described in Section 2.5.

### 2.11 Ubiquitination Level Validation Experiment

We collected protein precipitates from cells of each group according to experimental steps in Section 2.9, then carried out WB experiments following experimental steps in Section 2.5. In the WB experiment, RIPA lysis buffer containing 1% SDS was used. The protein precipitates were boiled at 95°C for 10 min for complete denaturation, diluted 10 times to reduce SDS concentration to 0.1%, and then immunoprecipitated with anti‐PLK1 antibody. The primary antibodies used included the K48‐linkage Specific Polyubiquitin antibody (#4289, Cell Signaling Technology, USA) in addition to the PLK1 antibody.

### 2.12 Immunofluorescence Detection

We collected transfected or drug‐treated OC cells, fixed them with 4% paraformaldehyde for 15 min, washed them three times with PBS, and then sequentially permeabilized them with 1% Triton X‐100 and blocked them with 10% bovine serum for 30 min to 2 h. Anti–γ H2AX primary antibody (AP0687, ABclonal, China) was added with a dilution ratio of 1:7000 and incubated overnight at 4°C. After washing, fluorescent‐conjugated secondary antibody (such as Alexa Fluor 488) was used and incubated at room temperature for 2 h. After PBS washing, the nuclei were stained with 4′, 6‐diamididinedione (DAPI). After sealing the slides, imaging was observed using a fluorescence microscope. Finally, the number of changes in the number of γ H2AX lesions was analyzed using ImageJ software.

### 2.13 Comet Assay

To analyze the level of DNA damage in cells, the Comet assay kit (catalog number KTA3040, Abbkine Scientific Co. Ltd., China) was utilized. In simple terms, we mixed the cells from each treatment group with 0.6% low‐melting‐point agarose. The mixture was put onto a comet slide, incubated in a dark environment at 4°C for 30 min, and immersed in a freshly prepared cold lysis solution. Finally, we performed horizontal electrophoresis of the slide at a rate of 1.0 V/cm and stained it with PI in the dark. Images were finally obtained using a fluorescence microscope (Leica DM 400B LED, Germany).

### 2.14 Data Preprocessing

All the experiments were repeated at least three times. Data were statistically analyzed using GraphPadPrism (GraphPad Software, USA), with results displayed as mean ± standard deviation. Comparisons between two groups were analyzed using Student's *t*‐test, while comparisons among multiple groups were analyzed using one‐way analysis of variance (ANOVA), followed by Tukey's post hoc test. Significance was set at *p* < 0.05.

## 3 Results

### 3.1 Upregulation of USP38 in OC Regulates the Malignant Behavior of Cancer Cells

**FIGURE 1:**
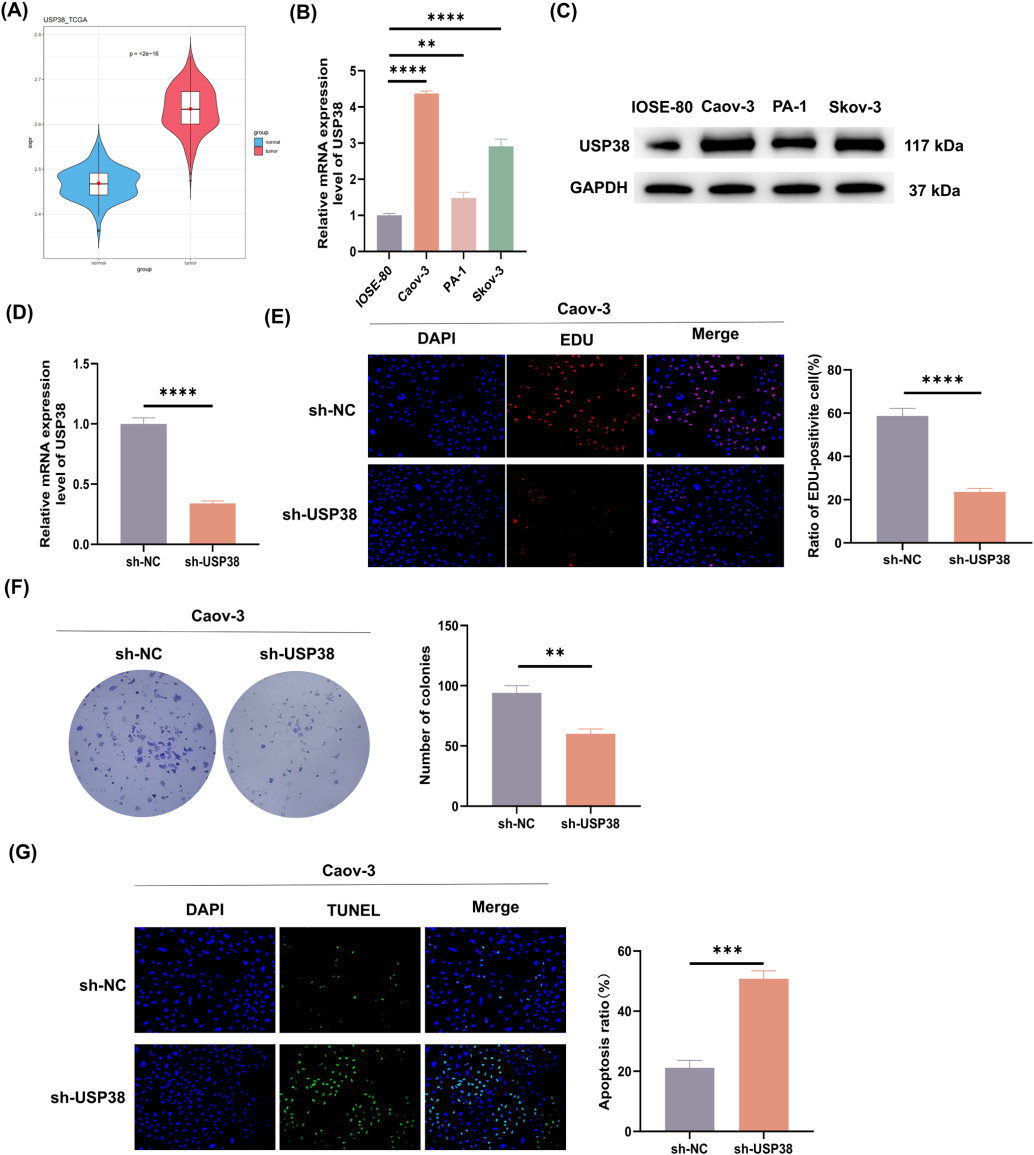
High expression of USP38 in OC mediates malignant progression. (A) Analysis of the expression level of USP38 in OC tissues and adjacent tissues based on TCGA‐OV database; (B) qPCR analysis of the mRNA expression level of USP38 in normal cell lines (IOSE‐80) and OC cell line (Caov‐3, PA‐1, Skov‐3), *n* = 3; (C) WB analysis of the protein expression level of USP38 in normal cell lines (IOSE‐80) and OC cell line (Caov‐3, PA‐1, Skov‐3); (D) qPCR analysis of the mRNA expression level of USP38 in Caov‐3 cells, *n* = 3; (E) EdU experiment analysis of the red fluorescence content of Caov‐3 cells in each group, *n* = 3; (F) Colony formation assay analyzed colony count of Caov‐3 cells in each group, *n* = 3; (G) TUNEL experiment analysis of the green fluorescence content of Caov‐3 cells in each group, *n* = 3; **p < 0.01, ***p < 0.001, ****p < 0.0001.

USP38 is proven to be differentially expressed in malignant tumors, thereby regulating the malignant progression of tumors [22, 24, 26]. This study probed into the relationship between USP38 and OC. We analyzed the expression level of USP38 in OC using the TCGA‐OV database and found that, compared to adjacent normal tissues, USP38 was greatly upregulated in OC tissues (Figure 1A). To validate the accuracy of the bioinformatics results, this study conducted cell experiments. We assessed the mRNA and protein expression of USP38 in normal ovarian epithelial cells (IOSE‐80) and OC cells (Caov‐3, PA‐1, Skov‐3) by utilizing qPCR and WB analysis. The results manifested that compared with normal ovarian epithelial cells, both mRNA and protein expression levels were significantly elevated in OC cells (Figure 1B,C). Due to the most significant differences between USP38 in Caov‐3 cells and IOSE‐80 cells, we chose the Caov‐3 cell line for the subsequent OC cell experiments. Combined with the bioinformatics analysis, cell experiments confirmed that USP38 was highly expressed in OC.

Next, we assessed the effect of USP38 knockdown on the malignant behavior of OC cells. First, we constructed USP38 overexpression groups based on the Caov‐3 cell line: the sh‐NC group and the sh‐USP38 group. We employed qPCR to detect transfection efficiency, finding that compared to the sh‐NC group, the mRNA expression level of USP38 was significantly reduced in the sh‐USP38 group (Figure 1D). Afterward, we used the EdU assay and colony formation assay to assess the proliferation of cells in each group, revealing that compared to the sh‐NC group, the red fluorescence intensity of cells in the sh‐USP38 group was dramatically reduced, and the number of colony formations was dramatically decreased (Figure 1E,F). This indicated that knocking down USP38 repressed the proliferation ability of OC cells. The detection of the apoptosis of cells in each group was examined by TUNEL assay, which demonstrated that the green fluorescence intensity in the sh‐USP38 group was significantly stronger than that in the sh‐NC group, indicating an elevation in the apoptosis rate in the sh‐USP38 group (Figure 1G). In summary, USP38 is highly expressed in OC cells, and silencing USP38 expression represses the proliferation of cancer cells and elevates the apoptosis rate of cancer cells.

### 3.2 USP38 Binds to PLK1, Mediates Deubiquitination of PLK1, and Stabilizes PLK1 Protein Expression

**FIGURE 2:**
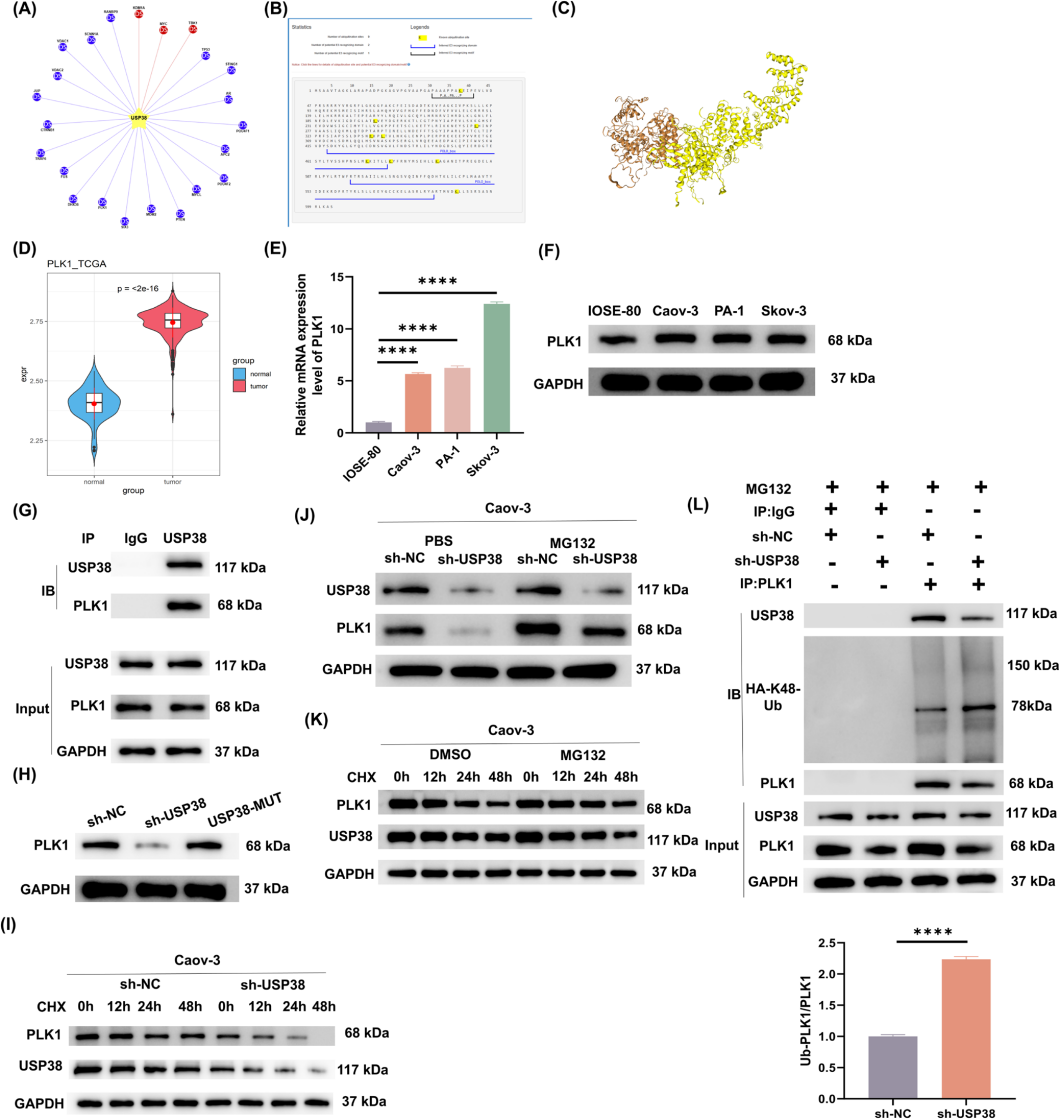
USP38 binds to PLK1 to mediate PLK1 deubiquitination. (A, B) Prediction of the downstream target proteins of USP38 and analysis of the deubiquitination site between USP38 and the target proteins based on the Ubibrowser database; (C) Prediction of the protein docking diagram of USP38 and PLK1 using the Hdock website; (D) Analysis of the expression of PLK1 in OC tissues and adjacent normal tissues based on the TCGA‐OV database; (E) Analysis of the mRNA expression levels of PLK1 in normal cell lines (IOSE‐80) and OC cell lines (Caov‐3, PA‐1, Skov‐3) using qPCR, *n* = 3; (F) Analysis of the protein expression levels of PLK1 in normal cell lines (IOSE‐80) and OC cell lines (Caov‐3, PA‐1, Skov‐3) using WB; (G) Analysis of the binding relationship between USP38 and PLK1 by CO‐IP experiment in Caov‐3 cells; (H) WB analysis of PLK1 protein expression levels in Caov‐3 cells from each group; (I) Caov‐3 cells in different groups treated with CHX, with the protein expression levels of USP38 and PLK1 analyzed at different times (0, 12, 24, and 48 h) using WB; (J) Caov‐3 cells in different groups treated with PBS or MG132, with the protein expression levels of USP38 and PLK1 detected by using WB; (K) Caov‐3 cells in different groups treated with CHX and MG132, with the protein expression levels of USP38 and PLK1 analyzed at different times (0, 12, 24, and 48 h) using WB; (L) Analysis of the ubiquitination level of PLK1 in different groups of Caov‐3 cells by HA‐K48‐Ub; ****p < 0.0001.

The above study demonstrated that USP38 regulated the malignant behavior of OC. We continued to further probe into the regulatory mechanism of USP38. USP38 is a common deubiquitinase that can bind target proteins and mediate protein stability [22, 27]. Based on this, we applied the Ubibrowser database to predict the downstream target proteins of USP38, unraveling that USP38 had a total of 23 target proteins (Figure 2A). Among them, PLK1 attracted our attention. Few reports on the research between PLK1 and USP38 are found, and most studies indicated that PLK1 can facilitate the malignant progression of OC [28, 29]. Then, we used the Ubibrowser database again to predict possible deubiquitination sites between USP38 and PLK1, discovering 9 deubiquitination sites between USP38 and PLK1 (Figure 2B). In addition, the protein docking map based on the Hdock website also demonstrated that USP38 had a binding relationship with PLK1 (Figure 2C). In conclusion, these results of bioinformatics indicated that USP38 bound to PLK1 and mediated the deubiquitination of PLK1.

Next, we analyzed the expression of PLK1 in OC cells. Analysis of the expression level of PLK1 in OC based on the TCGA‐OV database revealed that PLK1 was significantly highly expressed in OC tissues compared with adjacent normal tissues (Figure 2D). Next, this study applied cell experiments to verify the accuracy of the results from bioinformatics analysis. We used qPCR to analyze the mRNA expression levels of PLK1 in normal ovarian epithelial cells and OC cells and discovered that, compared to normal ovarian epithelial cells, the mRNA levels of PLK1 were significantly increased in OC cells (Figure 2E). WB detection also demonstrated that the protein expression level of PLK1 in OC cells was higher than that in normal ovarian epithelial cells (Figure 2F).

The above results verified that PLK1 was highly expressed in OC, indicating its regulatory role in OC. Subsequently, to confirm the protein regulatory role of USP38 on PLK1, we launched cell experiments. The results of the CO‐IP experiment between USP38 and PLK1 based on the Caov‐3 cell line demonstrated that the USP38 antibody immunoprecipitated PLK1 protein (Figure 2G), which indicated that there was a binding relationship between USP38 and PLK1. We hypothesize that the deubiquitinase activity of USP38 may play a crucial role in mediating the deubiquitination of PLK1. To test this hypothesis, we constructed a USP38 mutant, USP38‐MUT. The results of the WB experiment showed that sh‐USP38 reduced the protein expression of PLK1, while the expression level of PLK1 was relatively unaffected by USP38‐MUT (Figure 2H). This suggested that USP38 regulated PLK1 expression in a deubiquitinase activity‐dependent manner. To figure out the impact of USP38 on the stability of PLK1 protein, we designed related experiments. The USP38 abnormal expression groups: sh‐NC and sh‐USP38, were constructed based on the Caov‐3 cell line, and the protein synthesis inhibitor CHX was introduced into the cell culture medium. WB was employed to analyze the protein expression levels of USP38 and PLK1 in cells in each group at different times (0, 12, 24, and 48 h). The results showed that compared to sh‐NC, cells with USP38 knocked down had a significantly shorter half‐life of PLK1 protein after treatment with CHX, indicating that USP38 maintained the protein stability of PLK1 by repressing protein degradation (Figure 2I). In addition, we treated cells with PBS or the proteasome inhibitor MG132, revealing that MG132 rescued the downregulation of PLK1 protein by sh‐USP38, and thus confirming that the USP38‐mediated changes in PLK1 protein expression were achieved through the ubiquitin‐proteasome pathway (Figure 2J). To further confirm that USP38 stabilized the expression of PLK1 through the ubiquitin proteasome pathway, we supplemented the CHX treatment experiment with and without MG132. The experimental results showed that compared with the DMSO group, cells treated with MG132 showed a significant increase in the half‐life of PLK1 protein after CHX treatment, while the half‐life of USP38 showed no significant change, indicating that USP38 maintained the protein stability of PLK1 by inhibiting protein degradation (Figure 2k). Testing the ubiquitination level of PLK1 based on the abnormal expression grouping of USP38 uncovered that compared with the sh‐NC group, the ubiquitination level of PLK1 in the sh‐USP38 group was increased, and the protein level was decreased (Figure 2L). These results demonstrated that USP38 bound to PLK1 to mediate the deubiquitination of PLK1, thereby stabilizing the protein expression level of PLK1.

Taken together, USP38 binds to PLK1 in OC cells, mediating the deubiquitination of PLK1, thereby stabilizing the expression of PLK1 protein. PLK1 is highly expressed in OC.

### 3.3 USP38 Stabilizes the Protein Level of PLK1 to Boost DDR in OC Cells

**FIGURE 3:**
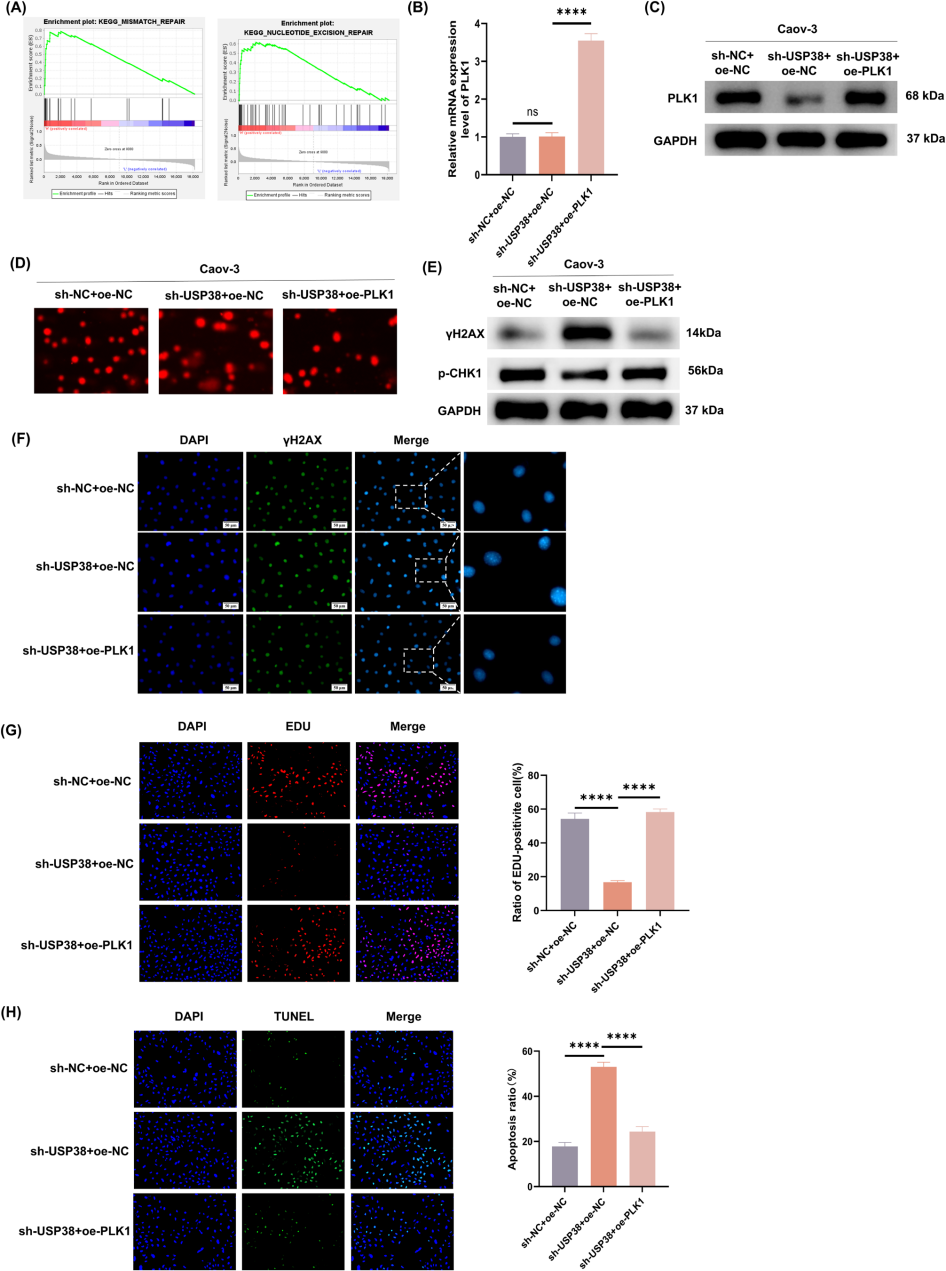
USP38 stabilizes the protein level of PLK1 to facilitate DDR in OC cells. Groups: sh‐NC + oe‐NC, sh‐USP38 + oe‐NC, sh‐USP38 + oe‐PLK1. (A) Analysis of the signaling pathways affected by differential expression of PLK1 using GSEA; (B) qPCR analysis of PLK1 mRNA expression levels in Caov‐3 cells from each group, *n* = 3; (C) WB analysis of PLK1 protein expression levels in Caov‐3 cells from each group; (D) Analysis of comet tail length and area in Caov‐3 cells from each group by comet assay, with the level of DNA damage evaluated; (E) WB analysis of γH2AX and p‐CHK1 protein expression in Caov‐3 cells from each group; (F) Immunofluorescence detection of the number of lesions of γH2AX in cells; (G) Analysis of the red fluorescence content in Caov‐3 cells from each group by EdU assay, *n* = 3; (H) Analysis of the green fluorescence content in Caov‐3 cells from each group by TUNEL assay, *n* = 3; ****p < 0.0001, ns indicates no significant difference.

The above experimental results verified that USP38 exerted the oncogenic effect on OC and suggested that USP38 mediated the stability of PLK1 protein. To further analyze the regulatory mechanism of USP38, we used GSEA for single‐gene enrichment analysis of PLK1 and found that the significant enrichment of DEGs caused by high‐ or low‐expression of PLK1 was in DDR‐related pathways, such as the MISMATCH_REPAIR pathway and NUCLEOTIDE_EXCISION_REPAIR pathway (Figure 3A). Given this, we believed that USP38 stabilized the protein level of PLK1 in OC, thereby boosting DDR in cancer cells and regulating the malignant behavior of cancer cells. To verify this hypothesis, we conducted relevant experiments. First, we established experimental groups based on Caov‐3 cells: sh‐NC + oe‐NC; sh‐USP38 + oe‐NC; sh‐USP38 + oe‐PLK1. Using qPCR to detect the mRNA expression level of PLK1 in cells in each group, we observed no great change in the mRNA level of the sh‐NC + oe‐NC group and the sh‐USP38 + oe‐NC group. The mRNA level of the sh‐USP38 + oe‐PLK1 group was significantly higher than that of the sh‐USP38 + oe‐NC group (Figure 3B). The WB detection on the protein expression levels of PLK1 in each group of cells demonstrated that compared to the sh‐NC + oe‐NC group, the protein level of PLK1 in the sh‐USP38 + oe‐NC group decreased, while the trend of decreased PLK1 protein level in the sh‐USP38 + oe‐PLK1 group was restored (Figure 3C). Then, we applied the comet experiment in the assessment of DNA damage of cells in each group. Compared with the sh‐NC + oe‐NC group, the length and area of the comet tail in the sh‐USP38 + oe‐NC group increased significantly, while the length and area of the comet tail in the sh‐USP38 + oe‐PLK1 group were restored (Figure 3D). The DNA damage response system involves the phosphorylation of histone variant H2AX (γH2AX), which plays a key role in maintaining genomic stability by mediating signal transduction and facilitating the repair of DNA double‐strand breaks [30]. It is shown that repressing PLK1 can lead to exacerbated DNA damage, a phenomenon that can be reflected by the upregulation of γH2AX levels [31]. The results of the WB experiment showed that compared with the sh‐NC + oe‐NC group, γH2AX expression was significantly increased in the sh‐USP38 + oe‐NC group, while it was restored in the sh‐USP38 + oe‐PLK1 group. The expression of p‐CHK1 showed an opposite trend to that of γH2AX (Figure 3E). The immunofluorescence results were consistent with the WB results; that is, compared with the sh‐NC + oe NC group, the number of γH2AX lesions in the sh‐USP38 + oe NC group significantly increased, while the number of γH2AX lesions in the sh‐USP38 + oe PLK1 group was restored (Figure 3F). This suggested that silencing USP38 facilitated DNA damage in OC cells, and overexpression of PLK1 reduced this enhancement. In addition, we also examined the proliferation and apoptosis of cells in each group using EdU and TUNEL assays, revealing that compared to the sh‐NC + oe‐NC group, the red fluorescence content of cells in the sh‐USP38 + oe‐NC group was considerably reduced, while the green fluorescence content was considerably increased. In the sh‐USP38 + oe‐PLK1 group, the changes in the two fluorescence contents were restored (Figure 3G,H). This indicated that silencing USP38 dampened the proliferation ability of OC cells and elevated the apoptosis rate, and overexpression of PLK1 weakened its regulatory effect on this basis.

To fully confirm the regulatory relationship between USP38 and PLK1, we constructed experimental groups based on Caov‐3 cells: sh‐NC + oe‐NC, sh‐USP38 + oe‐NC, sh‐USP38 + PLK1 WT, and sh‐USP38 + PLK1 KD. Compared with the sh‐NC + oe‐NC group, there was no significant change in PLK1 mRNA in the sh‐USP38 + oe NC group, while PLK1 was significantly increased or decreased in the sh‐USP38 + PLK1 + WT group and sh‐USP38 + PLK1 KD group (Figure S1A). The WB results showed that compared with the sh‐NC + oe NC group, the PLK1 protein in the sh‐USP38 + oe‐NC group was significantly reduced, while the PLK1 in the sh‐USP38 + PLK1 + WT group and sh‐USP38 + PLK1 KD group were significantly increased or decreased on this basis (Figure S1B). The comet experiment results showed that compared with the sh‐NC + oe NC group, the length and area of the comet tail in the sh‐USP38 + oe NC group significantly increased, while the length and area of the comet tail in the sh‐USP38 + PLK1 + WT group and sh‐USP38 + PLK1 KD group significantly decreased or increased on this basis (Figure S1C). WB detection of DNA damage markers also found that compared with the sh‐NC + oe NC group, the expression level of γ H2AX in the sh‐USP38 + oe NC group was significantly increased, while the expression of γ H2AX in the sh‐USP38 + PLK1 + WT group and sh‐USP38 + PLK1 KD group was significantly decreased or increased on this basis, while p‐CHK1 showed the opposite trend (Figure S1D). The immunofluorescence results were consistent with the WB results. Compared with the sh‐NC + oe NC group, the number of γ H2AX lesions in the sh‐USP38 + oe NC group significantly increased, while the number of γ H2AX lesions in the sh‐USP38 + PLK1 + WT group and sh‐USP38 + PLK1 KD group decreased or increased significantly (Figure S1E). The results of EDU and TUNEL experiments showed that compared with the sh‐NC + oe‐NC group, the red fluorescence content of cells in the sh‐USP38 + oe‐NC group was significantly reduced, and the green fluorescence was significantly increased. However, in the sh‐USP38 + PLK1 + WT group and sh‐USP38 + PLK1 KD group, the red fluorescence was significantly increased or decreased on this basis, and the green fluorescence was significantly decreased or increased on this basis (Figure S1F,G).

We constructed experimental groups based on Caov‐3 cells: oe‐NC + DMSO, oe‐USP38 + DMSO, and oe‐USP38 + PLK1 inhibitor. The experimental results showed that overexpression of USP38 enhanced the proliferation ability of ovarian cancer cells and reduced apoptosis rate. On this basis, inhibiting the expression of PLK1 weakened the regulatory effect of USP38 on cells (Figure S2A–G).

Collectively, USP38 in OC cells can bind to PLK1 to stabilize its expression and boost DDR in cancer cells, thereby enhancing the proliferation ability of cancer cells and reducing the apoptosis rate of cancer cells.

## 4 Discussion

This study demonstrated USP38 as a pro‐cancer factor in OC, which is highly expressed in OC. Silencing USP38 dampened the proliferation ability of cancer cells and increased the cancer apoptosis rate. Further analysis of its regulatory mechanism revealed that USP38 bound to PLK1, mediated deubiquitination of PLK1, and stabilized the protein expression level of PLK1. PLK1 was also highly expressed in OC, mediating cancer cell DDR‐related signaling pathways (MISMATCH_REPAIR pathway and NUCLEOTIDE_EXCISION_REPAIR pathway). Subsequent cell experiments confirmed that silencing USP38 reduced the expression level of PLK1 protein and inhibited the DDR capacity of OC cells, thereby reducing the proliferation ability of cancer cells and increasing the cancer apoptosis rate. Overexpression of PLK1 on the basis of silenced USP38 restored the regulatory effects conferred by silenced USP38. In conclusion, the deubiquitinase USP38 bound to PLK1 to stabilize PLK1 expression, boosting DDR in OC cells and facilitating cancer cell proliferation, thereby reducing the apoptosis rate of cancer cells.

As a member of the USP family, USP38 boosts protein stability and mediates cellular physiological activities by deubiquitinating target proteins [20]. We herein analyzed the impact of USP38 on the activity of OC cells. Cell experiments have confirmed that USP38 was highly expressed in OC tissues and cells. Silencing USP38 dampened the proliferation ability of cancer cells and potentiated their apoptosis ability. Similarly, Zhang et al. [32] pointed out in a study on lung adenocarcinoma (LUAD) that the expression of USP38 is up‐regulated in LUAD and the higher expression level of USP39 correlates with the worse prognosis. Silencing USP38 can repress the proliferation of LUAD cells in vitro and reduce the size and activity of xenograft tumors in mice. However, Zhan et al. [22] asserted that USP38 expression was down‐regulated in clinical CRC samples and cell lines. Silencing USP38 reinforces the proliferation of CRC cells in vitro and expands the size of xenografts in mice [22], indicating that the expression levels and functions of USP38 in different cancers are different and need to be analyzed specifically. The physiological activities of cells or organisms are complex and diverse, and different cancers have their own characteristics. Under such conditions, the expression level of USP38 is inevitably influenced by multiple factors. The role of USP38 in facilitating or repressing cancer may be linked with substrate proteins. This may be one of the reasons for the inconsistent expression and function of USP38 in different cancers. More reasons need to be explored continuously. In addition, considering the deubiquitination modification effect of USP38, we used bioinformatics tools to analyze USP38 binding proteins in OC. Among the 23 target proteins that bind to USP38, PLK1 has caught our attention. Cell experiments confirmed that PLK1 was highly expressed in OC. There was indeed a binding relationship between USP38 and PLK1 in OC, with 9 common sites for de‐ubiquitination. The view that PLK1 expression is upregulated in OC is consistent with the study of RödelF et al. [33]. However, studies on the relationship between USP38 and PLK1 are sparse in OC or other malignant tumors. Overall, this work can fill the gap in the field to identify new potential biomarkers for OC.

The above results demonstrated that USP38 was bound to PLK1 in OC. It is well known that USP38 binds substrate proteins, mediates deubiquitination of substrate proteins, and stabilizes their protein expression [27, 34]. We herein probed whether USP38 also played a role in deubiquitinating PLK1 and stabilizing its protein expression. Related cell experiments manifested that silencing USP38 enhanced the level of ubiquitination of PLK1 and reduced the level of PLK1 protein. Adding MG132 on the basis of silenced USP38 restored the declined protein levels of PLK1. This indicated that USP38 in OC bound to PLK1, mediating the deubiquitination of PLK1 and stabilizing the protein level of PLK1. Current research on PLK1 demonstrated that PLK1 is crucial for the precise regulation of cell division and maintenance of genomic stability in mitosis, spindle assembly, and DNA damage responses [35, 36]. The biological results of this project also proved that the differential expression of PLK1 was linked with DNA damage response‐related pathways. In addition, through cell experiments, we discovered that silencing USP38 reduced the protein level of PLK1 and weakened the DDR level of OC cells, thereby repressing the proliferation ability of cancer cells and potentiating the ability of cancer apoptosis. The regulatory effect was restored upon overexpression of PLK1 based on silencing USP38. This suggested that USP38 mediated DDR in OC cells by modulating the protein level of PLK1, thereby influencing the malignant behavior of cancer cells. Coincidentally, Lin et al. [37] asserted that treating GC cells with the PLK1 inhibitor AZD1775 can destroy the DDR ability of cancer cells and boost the antitumor activity of olaparib against GC. In these two studies, PLK1 is thus to be proven to regulate DDR in cancer cells, thereby mediating tumor progression, but we analyzed the regulatory mechanisms affecting its protein expression in OC.

In summary, USP38 can interact with PLK1 to stabilize PLK1 protein levels, thereby facilitating DDR in OC cells, boosting cancer cell proliferation, and reducing apoptosis levels. Our results confirmed the oncogenic effects of USP38 and PLK1 on OC and provided detailed validation of the relevant mechanism. Before this study, no scholars had dissected the relationship between USP38 and OC and its mechanisms. This investigation represents a major innovation in the field of OC research, filling a gap in this area. However, we did not design in vivo animal experiments to verify the hypothesis of the USP38/PLK1 axis enhancing DDR in OC cells, thereby leading to a lack of sufficient empirical support for the accuracy of the conclusions. We also failed to collect clinical samples for the detection of USP38 or PLK1 expression levels, and the project lacks certain clinical relevance. We expect to continue to study and resolve these issues in the future.

## Conflicts of Interest

The authors declare no conflicts of interest.

## Supporting information


**FIGURE S1:** Groups: sh‐NC + oe‐NC, sh‐USP38 + oe‐NC, sh‐USP38 + PLK1 WT, sh‐USP38 + PLK1 KD. (A) qPCR analysis of PLK1 mRNA expression levels in Caov‐3 cells from each group, *n* = 3; (B) WB analysis of PLK1 protein expression levels in Caov‐3 cells from each group; (C) Analysis of comet tail length and area in Caov‐3 cells from each group by comet assay, with the level of DNA damage evaluated; (D) WB analysis of γH2AX and p‐CHK1 protein expression in Caov‐3 cells from each group; (E) Immunofluorescence detection of the number of lesions of γ H2AX in cells; (F) Analysis of the red fluorescence content in Caov‐3 cells from each group by EdU assay, *n* = 3; (G) Analysis of the green fluorescence content in Caov‐3 cells from each group by TUNEL assay, *n* = 3; **p < 0.01, ***p < 0.001, ****p < 0.0001, ns indicates no significant difference.

**FIGURE S2:** Groups: oe‐NC + DMSO, oe‐USP38 + DMSO, oe‐USP38 + PLK1 inhibitor. (A) qPCR analysis of PLK1 mRNA expression levels in Caov‐3 cells from each group, *n* = 3; (B) WB analysis of PLK1 protein expression levels in Caov‐3 cells from each group; (C) Analysis of comet tail length and area in Caov‐3 cells from each group by comet assay, with the level of DNA damage evaluated; (D) WB analysis of γH2AX and p‐CHK1 protein expression in Caov‐3 cells from each group; (E) Immunofluorescence detection of the number of lesions of γ H2AX in cells; (F) Analysis of the red fluorescence content in Caov‐3 cells from each group by EdU assay, *n* = 3; (G) Analysis of the green fluorescence content in Caov‐3 cells from each group by TUNEL assay, *n* = 3; ***p < 0.001, ****p < 0.0001, ns indicates no significant difference.

## Data Availability

The data and materials in the current study are available from the corresponding author on reasonable request.
